# Restriction spectrum imaging with elastic image registration for automated evaluation of response to neoadjuvant therapy in breast cancer

**DOI:** 10.3389/fonc.2023.1237720

**Published:** 2023-09-15

**Authors:** Maren M. Sjaastad Andreassen, Stephane Loubrie, Michelle W. Tong, Lauren Fang, Tyler M. Seibert, Anne M. Wallace, Somaye Zare, Haydee Ojeda-Fournier, Joshua Kuperman, Michael Hahn, Neil P. Jerome, Tone F. Bathen, Ana E. Rodríguez-Soto, Anders M. Dale, Rebecca Rakow-Penner

**Affiliations:** ^1^ Department of Circulation and Medical Imaging, Norwegian University of Science and Technology, Trondheim, Norway; ^2^ Department of Oncology, Vestre Viken, Drammen, Norway; ^3^ Department of Radiology, University of California, San Diego, La Jolla, CA, United States; ^4^ Department of Bioengineering, University of California, San Diego, La Jolla, CA, United States; ^5^ Department of Radiation Medicine and Applied Sciences, University of California, San Diego, La Jolla, CA, United States; ^6^ Department of Surgery, University of California, San Diego, La Jolla, CA, United States; ^7^ Department of Pathology, University of California, San Diego, La Jolla, CA, United States; ^8^ Department of Physics, Norwegian University of Science and Technology, Trondheim, Norway; ^9^ Department of Radiology and Nuclear Medicine, St. Olav’s University Hospital, Trondheim, Norway

**Keywords:** breast cancer, locally-advanced breast cancer, neoadjuvant therapy, magnetic resonance imaging, breast MRI, diffusion-weighted imaging, restriction spectrum imaging

## Abstract

**Purpose:**

Dynamic contrast-enhanced MRI (DCE) and apparent diffusion coefficient (ADC) are currently used to evaluate treatment response of breast cancer. The purpose of the current study was to evaluate the three-component Restriction Spectrum Imaging model (RSI_3C_), a recent diffusion-weighted MRI (DWI)-based tumor classification method, combined with elastic image registration, to automatically monitor breast tumor size throughout neoadjuvant therapy.

**Experimental design:**

Breast cancer patients (*n=*27) underwent multi-parametric 3T MRI at four time points during treatment. Elastically-registered DWI images were used to generate an automatic RSI_3C_ response classifier, assessed against manual DCE tumor size measurements and mean ADC values. Predictions of therapy response during treatment and residual tumor post-treatment were assessed using non-pathological complete response (non-pCR) as an endpoint.

**Results:**

Ten patients experienced pCR. Prediction of non-pCR using ROC AUC (95% CI) for change in measured tumor size from pre-treatment time point to early-treatment time point was 0.65 (0.38-0.92) for the RSI_3C_ classifier, 0.64 (0.36-0.91) for DCE, and 0.45 (0.16-0.75) for change in mean ADC. Sensitivity for detection of residual disease post-treatment was 0.71 (0.44-0.90) for the RSI_3C_ classifier, compared to 0.88 (0.64-0.99) for DCE and 0.76 (0.50-0.93) for ADC. Specificity was 0.90 (0.56-1.00) for the RSI_3C_ classifier, 0.70 (0.35-0.93) for DCE, and 0.50 (0.19-0.81) for ADC.

**Conclusion:**

The automatic RSI_3C_ classifier with elastic image registration suggested prediction of response to treatment after only three weeks, and showed performance comparable to DCE for assessment of residual tumor post-therapy. RSI_3C_ may guide clinical decision-making and enable tailored treatment regimens and cost-efficient evaluation of neoadjuvant therapy of breast cancer.

## 1 Introduction

Neoadjuvant therapy of breast cancer is used to enable breast-conserving surgery, to provide an *in vivo* drug-sensitivity test bed (1, 2), and provide short- and long-term prognostic information. The goal of neoadjuvant therapy is pathological complete response (pCR), defined as no remaining tumor tissue in breast and lymph nodes as measured by post-surgical pathology, and which is associated with prognostic benefits such as improved survival and reduced recurrence rates (3). Early assessment of treatment response is important for tailoring treatment regimens for the best patient outcome, specifically identifying poor responders that are candidates for escalated treatment.

The current gold standard for neoadjuvant treatment response assessment in breast cancer is change in tumor size on dynamic contrast-enhanced MRI (DCE), manually assessed by the longest diameter in three dimensions (4). Changes in size may take several weeks before being detected by DCE, potentially delaying critical clinical decisions as well as requiring the administration of Gadolinium-based exogenous contrast agents. Furthermore, DCE-based manual measurements have conflicting results regarding residual cancer detection specificity (5) and require expert radiologist readers to delineate tumor tissue at each time point.

One MRI modality that does not require an exogenous contrast agent is diffusion-weighted MRI (DWI), a method that is sensitized to the microscopic diffusion of water molecules (6). In oncology, DWI has received increased recognition for its usefulness in detecting malignant tumors by reduced apparent diffusion coefficient (ADC), commonly associated with the restricted diffusion caused by highly cellular tumors (7). In the neoadjuvant therapy setting, several studies have indicated that an increase in ADC might predict treatment response (8–13), hypothesized to be caused by a reduction in cellularity through the course of therapy. However, despite the more restricted diffusion, untreated tumor ADC values have somewhat surprisingly been proven to be higher than that of healthy breast tissue (14). One possible reason is the existence of edema and necrosis, which results in a decrease in hindrance in extracellular water, increasing the ADC (15–17). Additionally, areas of hyper-restricted diffusion signal from healthy fatty tissue that lowers the ADC can make it challenging to differentiate lesions from normal healthy breast tissue by ADC alone. Consequently, the assessment of treatment response using ADC often requires time-consuming manual delineation of tumors to avoid the inclusion of any surrounding healthy breast tissue. This calls for the exploration of alternative techniques that maximize the potential of DWI as an adjunct or alternative to DCE methods.

Restriction Spectrum Imaging (RSI) is a multi-component modeling framework that uses DWI signal over broad ranges of diffusion weightings (*b*-values) to capture the restricted diffusion of intracellular water (18, 19). RSI estimates of cellularity are shown to be directly related to histopathological tumor cellularity in preclinical models (20) and Gleason grade in the human prostate (21, 22). Additionally, RSI is effective for treatment response assessment for glioma (18, 23) and has decreased sensitivity to edema compared to ADC (15). In the breast, a three-component RSI model (RSI_3C_) has been shown to improve tumor conspicuity and tumor discrimination from healthy breast tissue compared to ADC in untreated patients (14, 24) but has not yet been evaluated for treatment response assessment. The current study aimed to assess the ability of RSI_3C_ to both assess early response to treatment and evaluate post-therapy residual cancer compared to conventional manual DCE delineation, and subsequent DWI quantitation using ADC, both of which rely on extensive radiologist input.

## 2 Materials and methods

### 2.1 Subject eligibility

Twenty-seven breast cancer patients (median age 47 years, range 20-68) were included in this retrospective analysis from participants in a prospective phase II clinical trial; see **Table 1** for patient characteristics details. Criteria for inclusion in the trial included biopsy-proven (core needle) unilateral invasive breast cancer ≥2.5 cm (defined on imaging/clinical examination) with an indication for neoadjuvant therapy. We included all participants (*n*=31) from the University of California San Diego (UCSD) site who underwent multi-*b*-value DWI acquisition between December 2015 and June 2019. Written informed consent was obtained from all patients. The study was approved by the local institutional review board and conducted in accordance with the Declaration of Helsinki. Four patients were excluded from further analyses due to poor image quality for DCE (*n*=3) and DWI (*n*=1), resulting in 27 included patients. The sample size was determined by the maximum number of participants recruited at the time of analysis. The primary treatment was paclitaxel (+/-) experimental agent followed by anthracyclines.

**Table 1:** Clinical characteristics of patient cohort.

No. Patients	27
**Median patient age, years (range)**	47 (20-68)
Lesion type mass (mass vs. NME)	
Mass	24
Mass + NME	3
Histologic type	
NST	24
Metaplastic carcinoma	2
Mixed IDC/ILC	1
MBR score	
1	1
2	11
3	15
ER status	
Positive	15
Negative	12
PR status	
Positive	13
Negative	14
HER2 status	
Positive	3
Negative	23
Not analyzed	1
pCR status	
pCR	10
non-pCR	17
Median time from therapy start to MRI scan, days (range)	
Early-treatment	19 (15-26)
Mid-treatment	81 (48-94)
Post-treatment	153 (127-190)

NME, non-mass enhancement; NST, invasive breast cancer of no special type; IDC, invasive ductal carcinoma; ILC, invasive lobular carcinoma; MBR, Modified Bloom-Richardson; ER, estrogen receptor; PR, progesteron receptor; HER2, Human Epidermal Growth Factor Receptor 2; pCR, pathological complete response.

**Figure 1:**
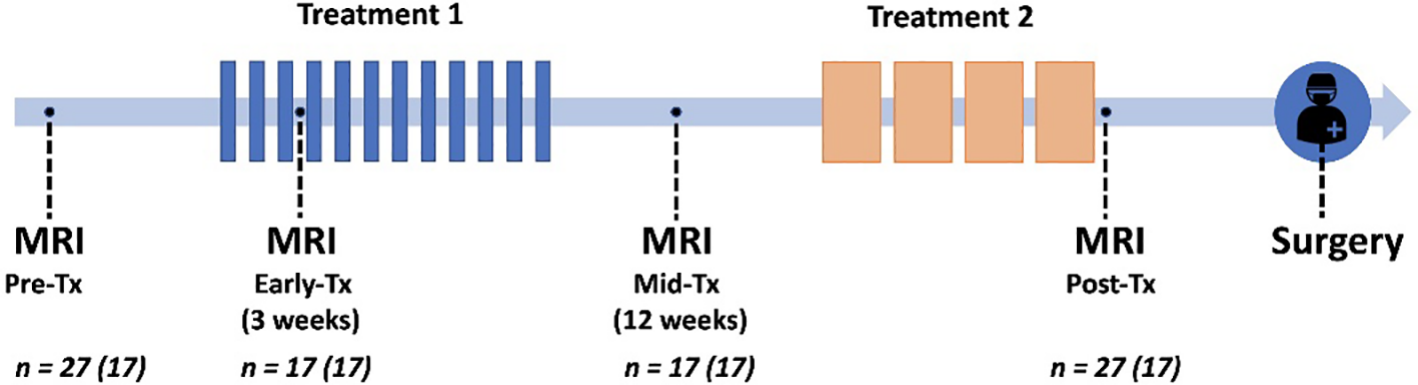
Trial schematic showing neoadjuvant treatments in relation to pre-treatment, early-treatment, mid-treatment, and post-treatment MRI, followed by surgery. Seventeen patients were scanned at all four time points (given in parenthesis in figure).

Patients underwent MRI at four time points to evaluate response to treatment: pre-treatment, early-treatment (3 weeks, range 2-4 weeks), mid-treatment (12 weeks, range 7-13 weeks), and post-treatment (22 weeks, range 18-27), illustrated in **Figure 1**. Out of the 27 patients, 17 patients received all four scans; for three patients, scans at specific time points were excluded due to major patient movement (*n*=1) and poor DWI image quality (*n*=2). This led to the following numbers available for analysis; pre-treatment (*n*=27), early-treatment (*n*=17), mid-treatment (*n*=17), and post-treatment imaging (*n*=27). Note that for five patients, surgery was performed directly after the mid-treatment time point, and this was thus categorized as a post-treatment scan rather than a mid-treatment. The pre-treatment scans (*n*=27) were previously used for the development of RSI_3C_ in two studies (14, 24).

### 2.2 MRI acquisition and image preprocessing

MRI data were acquired on a 3T GE scanner (MR750, DV25-26, GE Healthcare, Milwaukee, US) with an 8-channel breast array coil with a bilateral axial imaging plane. The MRI protocol included Gadolinium DCE (Gadovist or MultiHance), non-fat-saturated T_1_, and multi-*b*-value DWI acquisition. DCE acquisition parameters included TE = 2.6 ms, TR = 5.4 ms, flip angle = 10°, acquisition matrix 512 x 406, reconstruction matrix 512 x 512, and voxel size 0.625 x 0.625 (in-plane) x 2.4 (slice) mm^3^. DWI was performed using reduced field of view (FOV) echo-planar imaging (EPI) including the following parameters: SPECtral Inversion At Lipid (SPECIAL) fat suppression, TE = 82 ms, TR = 9000 ms, *b*-values (number of diffusion directions) = 0, 500 (6), 1500 (6), and 4000 (15) s/mm^2^, FOV = 160 x 320 mm^2^, acquisition matrix = 48 x 96, reconstruction matrix = 128 x 128, voxel size = 2.5 x 2.5 x 5.0 mm^3^, phase-encoding (PE) direction anterior to posterior (A/P).

All data analysis was performed using MATLAB 2020b (MathWorks, Natick, MA). DWI data were averaged across diffusion directions for each *b*-value, corrected for eddy current artifacts, motion (18), and geometric distortion (25), and resampled to match the geometry of the DCE images.

Fast longitudinal image registration (FLIRE) (26) was used to co-register DWI data to non-fat-saturated T_1_ and to longitudinally register all images and regions of interest (ROIs) to the pre-treatment time point. FLIRE is based on a well-established non-rigid deformable brain registration technique (27), which when optimized for the breast has been shown in preliminary studies to outperform existing registration methods, with significantly reduced run time (26). We provided four example cases to demonstrate differences in unregistered and registered images in supplemental data (**Supplemental Figures 1–4**).

### 2.3 Tumor assessment by DCE

To provide standard-of-care response assessment, the longest diameter of cancer in any plane (in cm) corresponding to biopsy-proven cancer was manually defined on post-contrast DCE by a breast radiologist (RRP) for each time point. For cases with several cancer lesions, the largest conglomerate of connected lesions was used as the definition of cancer.

### 2.4 Automatic tissue classification and tumor measurement using RSI_3C_

Full-lesion cancer and control regions of interest (ROIs) were manually defined at the pre-treatment time point on a high *b*-value DWI image (*b* = 1500 or 4000 s/mm^2^) avoiding macroscopic areas of necrosis, validated by a breast radiologist (RRP) as described in prior publications (14, 24). Cancer ROIs were drawn for the lesions corresponding to biopsy-proven cancer; for cases with several cancer lesions, the largest conglomerate of connected lesions was defined as the cancer ROI. Control ROIs were defined using a semi-automatic approach with the aim to include all representative healthy breast tissue; first, a rectangular box was placed around the entire healthy contralateral breast (only unilateral breast cancer was included in this study), then the background was masked using intensity thresholding and 3D connected components.

The DWI signal for all voxels across all available *b*-values was fitted to a previously-developed RSI_3c_ model (14, 24), given as:


$$S_{DWI}\left(b\right)=\;C_{1}\exp\left(-b\cdot ADC_{1}\right)+C_{2}\exp\left(-b\cdot ADC_{2}\right)+\;C_{3}\exp\left(-b\cdot ADC_{3}\right)$$

where *S_DWI_* is the diffusion signal in arbitrary units, *b* is the *b*-value in s/mm^2^, and C_i_ denotes the voxel-wise, unit-less signal contribution of each component *i*. The apparent diffusion coefficient (ADC_i_) values, given in mm^2^/s, were fixed across voxels as previously reported (17). C_1_ relates to restricted or hyperrestricted diffusion in cancer and healthy fatty tissue and C_2_ to hindered diffusion in cancer and healthy fibroglandular tissue, while C_3_ corresponds to fast diffusion and vasculature (17). The data were normalized to the 95^th^ percentile of the intensity of the computed geometric mean of C_1_ and C_2_ of the control ROI for each patient.

To create a global RSI_3C_ tissue classifier applicable across patients and time points, the first two components of RSI_3C_ (C_1_ and C_2_) were selected, as these have previously demonstrated excellent discrimination of cancer from healthy breast tissue (16). Joint C_1_ and C_2_ probability density functions (PDFs) for voxels in cancer and control ROIs were calculated for all patients simultaneously at the pre-treatment time point. The PDFs generated a lookup table of the posterior probability of cancer, given C_1_ and C_2_ measurements for any voxel. This was used to create voxel-wise probability maps for each individual patient at each time point (RSI_3C_ map).

**Figure 2:**
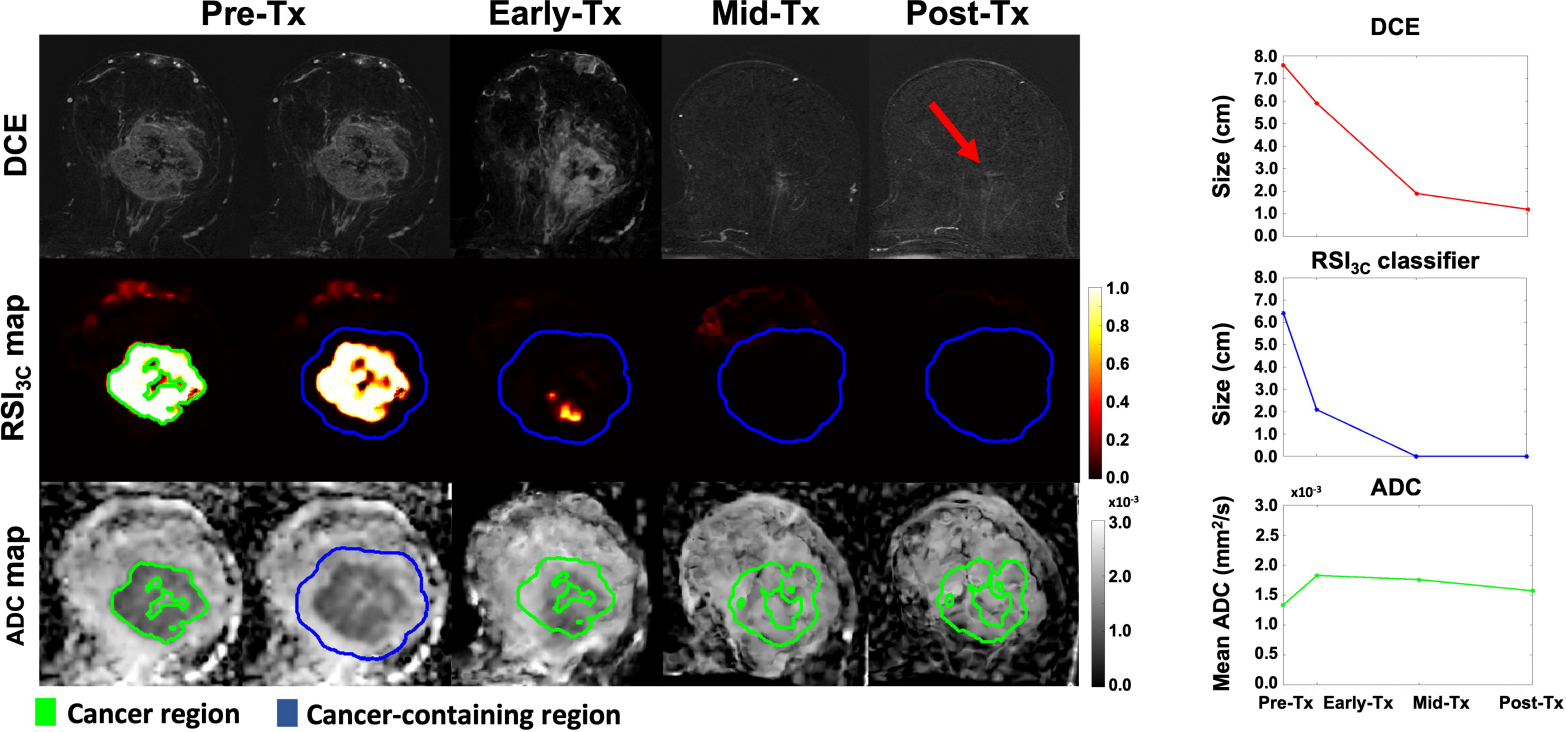
DCE, RSI_3c_ maps, and ADC maps with corresponding size (manual DCE measurement and RSI_3C_ classifier) and mean calculation (ADC) for all four time points for a subject with no remaining tumor tissue on final post-surgical pathology. The cancer region (green outline) at the pre-treatment time point was uniformly expanded by 1 cm to generate a cancer-containing region (blue outline). The longest diameter of cancer (in cm) was manually defined on post-contrast DCE for each time point. For the RSI_3C_ classifier, the largest single connected component within the cancer-containing region was identified and the longest diameter (in cm) was automatically assessed. To account for tumor growth outside of the tumor-containing region, any components connected to the tumor-containing region above a threshold of 0.5 were included in the analysis. The tumor-containing region at pre-treatment was applied for all subsequent registered time points. The mean ADC was calculated within the cancer region (green outline) for each time point. The RSI_3C_ classifier shows a more pronounced size decrease at the early-treatment time point compared to manual measurement by DCE. The RSI_3C_ classifier was more specific at the post-treatment time point, while there was still some remaining tumor left within the tumor bed at the post-treatment time point for the DCE (red arrow). Also, note that the RSI_3C_ classifier is well-defined within the cancer ROI at the pre-treatment time point (green outline). *Tx, treatment; DCE, dynamic contrast-enhanced MRI; RSI_3C_, three-component Restriction Spectrum Imaging model; ADC, apparent diffusion coefficient*.

**Figure 3:**
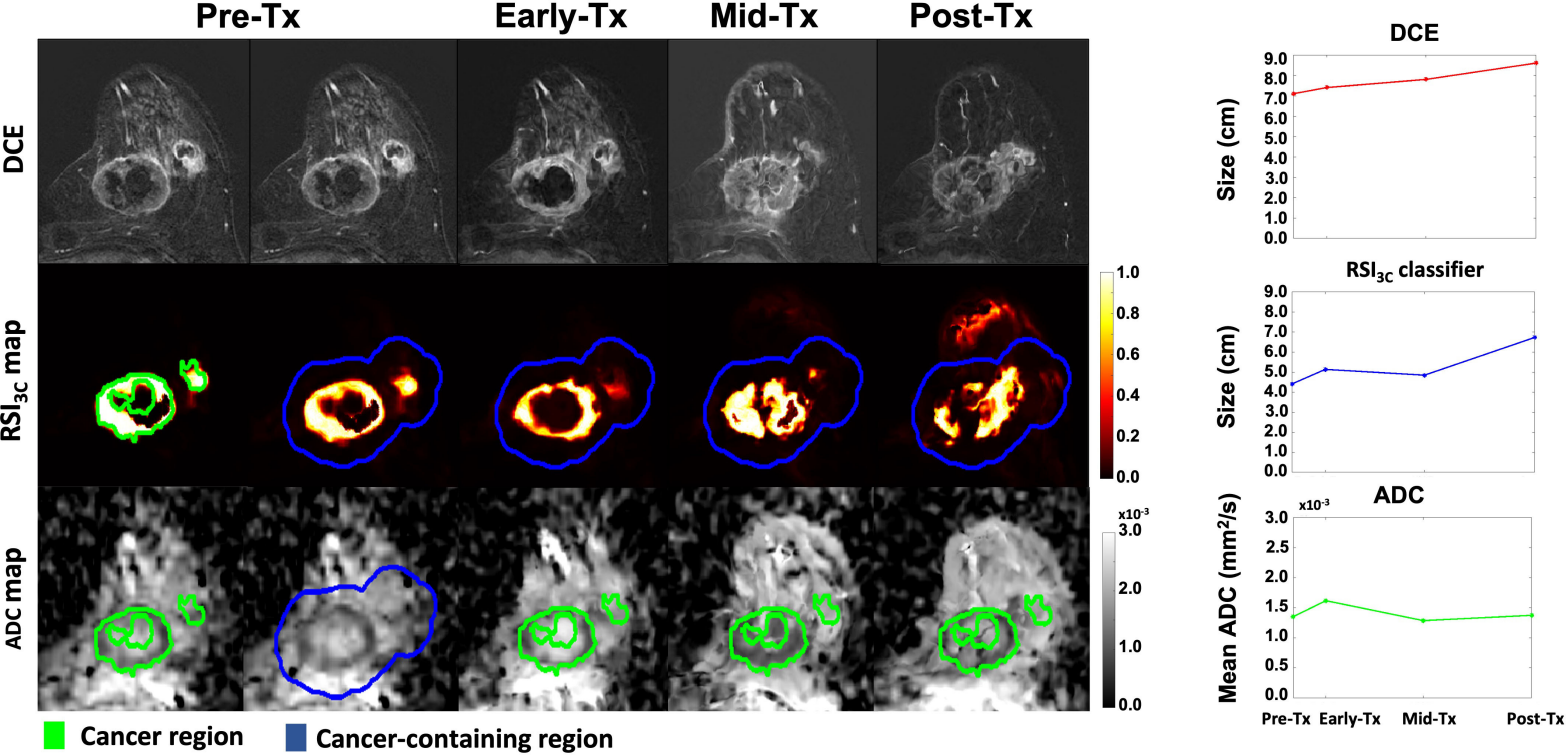
DCE, RSI_3c_ maps, and ADC maps with corresponding size (manual DCE measurement and RSI_3C_ classifier) and mean calculation (ADC) for all four time points for a non-responding subject with remaining tumor tissue on final post-surgical pathology. *Tx, treatment; DCE, dynamic contrast-enhanced MRI; RSI_3C_, three-component Restriction Spectrum Imaging model; ADC, apparent diffusion coefficient*.

To estimate the longest tumor dimension after voxel classification, the defined cancer ROI at the pre-treatment time point was uniformly expanded by 1 cm to generate a ‘tumor-containing region’ and used for analysis on the RSI_3C_ map (**Figure 2**). In addition, to account for any tumor growth outside of the tumor-containing region, any components connected to the tumor-containing region above 0.5 were included in the tumor-containing region. After the tumor-containing region was defined, the largest single connected component within the tumor-containing region above a 0.5 threshold on the RSI_3C_ map was identified. For this lesion, the longest diameter in any plane (in cm) was automatically calculated by using voxel coordinates; a detailed description of lesion size calculation is given in supplemental materials (**Supplementary Methods**). A one-dimensional RSI_3C_ measurement was chosen to ensure direct comparability with the longest tumor diameter manually defined on DCE. For two cases, any enhancement from the skin was masked as the focus of this study was the primary tumor. The tumor-containing region definition at pre-treatment was applied across all time points, thus limiting the manual definition of cancer ROI and semi-automatic definition of control ROI to the pre-treatment time point. An example of a non-responding subject is given in **Figure 3**.

### 2.5 Diffusion quantification using ADC

Conventional apparent diffusion coefficient (ADC) maps were calculated as described by Jensen et al. (22) using *b*-values< 1000 s/mm^2^ taken from the multi-*b*-value RSI_3C_ acquisition. The mean ADC value was assessed within the pre-treatment cancer ROI applied to all subsequent registered time points. The cancer ROI was used rather than the tumor-containing region (as for RSI_3C_) to avoid the inclusion of any healthy breast tissue at baseline (pre-treatment time point). This approach thus avoids the time-consuming and technically difficult manual delineation of a tumor undergoing treatment and ensures the same number of analyzed voxels for each time point. Any undefined values (zero and infinite) at *b* = 0 s/mm^2^ and ADC were excluded (no undefined values were present in the cancer ROI). Example cases including violin plots to display ADC distribution are included in supplemental materials (**Supplemental Figures 5, 6**).

### 2.6 Clinical response definition

The primary endpoint was non-pathological complete response (non-pCR). pCR was defined as no residual invasive disease with or without ductal carcinoma *in situ* in either breast or axillary lymph nodes after neoadjuvant therapy (ypT0/is, ypN0) (28). Assessment of pCR-status was performed on the post-surgical histological specimen, and patients were categorized into pCR and non-pCR groups. Non-pCR was used as endpoint rather than pCR as we argue it is more clinically relevant to identify non-responders that are candidates for escalated treatment. The post-treatment size and residual tumor cellularity (manually estimated) of the post-surgical specimen were recorded.

### 2.7 Assessment of treatment response by imaging metrics

The tumor size from DCE and RSI_3C_ classifier measurements, as well as mean ADC values, were analyzed for all patients (*n*=27) at the post-treatment time point. Absolute values were used as the purpose was to investigate the association with final pathology (non-pCR) at the post-treatment time point. In addition, for the patients who underwent all four scans (*n*=17), response to treatment during the course of treatment was assessed using the relative change in measured diameter sizes (ΔRSI_3C_, ΔDCE) and change in mean ADC (ΔADC) from pre-treatment to each of the early, mid, and post-treatment time points. The relative change was used to assess how change in imaging modality over time could predict non-pCR.

### 2.8 Statistical analysis

MATLAB 2020b (MathWorks, Natick, MA) and Excel Version 16.74 were used for statistical analysis. The area under the curve (AUC) of receiver operating characteristics (ROC) curves were calculated for all cases (*n*=27) at the post-treatment time point for DCE, RSI_3C_ classifier, and mean ADC to detect non-pCR (i.e. positive was defined as a patient with remaining tumor tissue, and so accurate detection of pCR corresponded to a negative classification in the imaging). Furthermore, for cases with all four scans (*n*=17), ROC curves were assessed for the ability of ΔRSI_3C_, ΔDCE, and ΔADC to predict non-pCR at the early, mid, and post-treatment imaging time points. We used an *a priori* assumption that an increase in mean ADC (8–13) and a decrease in RSI_3C_ classifier and DCE size represents response to treatment, in line with previous experience. Sensitivity, specificity, and accuracy were calculated for the threshold yielding the highest accuracy; for completeness, accuracy (acc_90_) and sensitivity (sens_90_) requiring specificity ≥ 90% were included in **supplemental materials** (**Supplemental Tables 1, 2**). Diagnostic sensitivity and specificity between techniques were compared by McNemar’s test, while DeLong’s test was used to compare ROC curves. Alpha was set to 0.025 due to correction for two primary outcomes (AUC and sensitivity/specificity).

## 3 Results

**Figure 4:**
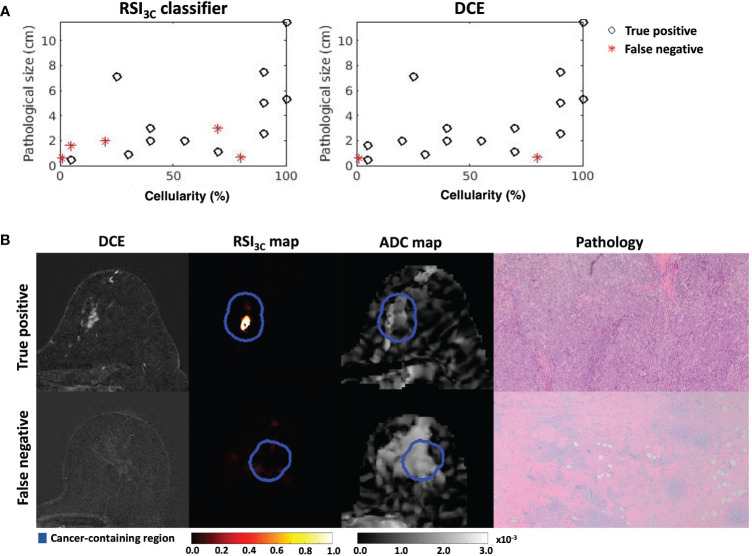
**(A)** Cases with remaining tumor tissue on final post-surgical pathology are included for the RSI_3c_ classifier and manual measurement by DCE. RSI_3C_ classifier has more false negative plots than DCE, with 3/5 cases associated with low cellularity. **(B)** Two example cases, where (upper row) a true positive case for both RSI_3C_ classifier (1.5 cm) and manual DCE (2.0 cm) had corresponding high cellularity of 70% (size 1.1 x 0.6 cm) on final post-surgical pathology, while (lower row) a false negative for RSI_3C_ classifier (0 cm) and manual DCE (0 cm) had low cellularity of 1% and similar size (0.6 x 0.5 cm) on final post-surgical pathology. ADC maps are displayed as a reference. *DCE, dynamic contrast-enhanced MRI; RSI_3C_, three-component Restriction Spectrum Imaging model; ADC, apparent diffusion coefficient*.

After post-surgical histology, 10/27 (37%) patients were classified as showing pCR. Of the 17 non-pCR patients, the RSI_3C_ classifier correctly identified 12 using a threshold of 0.75 cm for non-pCR definition, with 5 false negatives that showed generally small remaining tumor size and varied cellularity (**Figure 4A**). There was 1 false positive. Correspondingly, DCE correctly classified 15 non-pCR patients, with only 2 false negatives and 3 false positives (using a threshold of 0.60 cm for non-pCR definition). Example classifications are shown in **Figure 4B**. Correlation plots between tumor size by RSI_3C_ classifier and DCE and cellularity are included in supplementary materials (**Supplemental Figure 7**) and display moderate correlation.

Results for the post-treatment time point are given in **Table 2**; corresponding ROC curves are given in **Supplemental Figure 8**. Sensitivity, specificity, and accuracy for absolute post-treatment tumor size were 0.88, 0.70, and 0.81 for DCE, and were 0.71, 0.90, and 0.78 for the RSI_3C_ classifier, and mean ADC at post-treatment gave 0.76, 0.50 and 0.67.

**Table 2:** Sensitivity, specificity, accuracy, and receiver operating characteristics (ROC) area under the curve (AUC) for prediction of non-pCR for manual dynamic contrast-enhanced MRI (DCE), three-component Restriction Spectrum Imaging model (RSI_3C_) classifier and the mean apparent diffusion coefficient (ADC) after all neoadjuvant therapy prior to surgical intervention (post-Tx time point).

	DCE	RSI_3C_ classifier	ADC
Threshold value	0.60 cm	0.75 cm	1.5 × 10^-3^ mm^2^/s
Sensitivity (95% CI) Post-Tx	0.88 (0.64-0.99)	0.71 (0.44-0.90)	0.76 (0.50-0.93)
Specificity (95% CI) Post-Tx	0.70 (0.35-0.93)	0.90 (0.56-1.00)	0.50 (0.19-0.81)
Accuracy (95% CI) Post-Tx	0.81 (0.61-0.94)	0.78 (0.58-0.91)	0.67 (0.46-0.83)
ROC AUC	0.79	0.80	0.52

pCR, pathological complete response; Tx, treatment. For context, the longest diameter at pre-Tx was on average 4.9 cm (+/- 2.5) for DCE and 3.6 cm (+/- 1.7) for RSI_3C_. Mean ADC at pre-Tx was 1.1 × 10^-3^ mm^2^/s (+/- 0.3 × 10^-3^).

McNemar’s test for comparison of sensitivity and specificity did not show significant differences for comparison between any modalities.

The AUC of the ROC evaluating the change in measured tumor size from pre-treatment to the early-treatment, mid-treatment, and post-treatment time points were 0.64, 0.71, and 0.80 for ΔDCE; 0.65, 0.60, and 0.76 for the ΔRSI_3C_; and 0.45, 0.35 and 0.36 for ΔADC (under the assumption that ADC increases with response). **Table 3** shows the complete data including threshold values; corresponding ROC curves are given in **Supplemental Figure 9**. DeLong’s test for comparison of ROC curves for early-treatment time point resulted in non-significant p-values throughout: ΔDCE vs. ΔRSI_3C_ p=0.94, ΔDCE vs. ΔADC p=0.31, ΔRSI_3C_ vs. ΔADC p=0.43, mid-treatment time point: ΔDCE vs. ΔRSI_3C_ p=0.42, ΔDCE vs. ΔADC p=0.10 and ΔRSI_3C_ vs. ΔADC p=0.14, and post-treatment: ΔDCE vs. ΔRSI_3C_ p=0.66, ΔDCE vs. ΔADC p=0.03, and ΔRSI_3C_ vs. ΔADC p=0.04.

**Table 3:** Sensitivity, specificity, accuracy, and receiver operating characteristics (ROC) area under the curve (AUC) for the performance of ΔDCE, ΔRSI_3C,_ and ΔADC for prediction of non-pCR at each time point.

		ΔDCE	ΔRSI_3C_	ΔADC (↑ = response)
Early-Tx (3 weeks)	AUC (95%CI) Sens. Spec. Accu. Thresh.	0.64 (0.36-0.91) 0.55 1.00 0.71 -0.07	0.65 (0.38-0.92) 0.91 0.50 0.76 -0.58	0.45 (0.16-0.75) 0.82 0.33 0.65 0.47
Mid-Tx (12 weeks)	AUC (95%CI) Sens. Spec. Accu. Thresh.	0.71 (0.45-0.96) 0.91 0.33 0.71 -0.73	0.60 (0.32-0.88) 1.00* 0.00* 0.65* <-1.00*	0.35 (0.06-0.64) 0.91 0.17 0.65 1.06
Post-Tx	AUC (95%CI) Sens. Spec. Accu. Thresh.	0.80 (0.59-1.00) 0.73 0.83 0.76 -0.72	0.76 (0.52-0.99) 0.64 1.00 0.76 -0.81	0.36 (0.07-0.65) 0.91 0.17 0.65 0.99

Note that the post-Tx time point is after all neoadjuvant therapy prior to surgical intervention. As the data is normalized to pre-Tx time point, threshold values are unitless multiplication factors.

* The optimal accuracy is achieved by a threshold where all cases are classified as non-pCR (sensitivity = 100%).

pCR, pathological complete response; Tx, treatment; ΔDCE, change in size from pre-treatment time point for manual dynamic contrast-enhanced MRI; ΔRSI_3C_, change in size from pre-treatment time point for the three-component Restriction Spectrum Imaging model classifier; ΔADC, change in mean value from pre-treatment time point for apparent diffusion coefficient.

## 4 Discussion

Our study shows that the classifier based on automatic cancer tissue detection using a three-component Restriction Spectrum Imaging model (RSI_3C_) indicated prediction of response to treatment after only three weeks (AUC = 0.65, 95%CI 0.38-0.92). Further, the RSI_3C_ classifier could identify 71% of cases that corresponded to residual tumor at surgery with 90% specificity in the later phase of treatment, similar to the performance by manual tumor measurement on DCE (0.65% sensitivity with 90% specificity). In contrast to a conventional workflow using DCE or ADC, which requires manual user input in the form of ROIs or tumor diameter measurement, the RSI_3C_ classifier is automatic beyond the pre-treatment MRI scan. The findings suggest that the RSI_3C_ classifier is sensitive to early time point changes and provides adequate classification at the post-treatment stage, supporting the role of the RSI_3C_ classifier to automatically monitor breast tumor size throughout neoadjuvant therapy.

The performance, indicating prediction of response, was present already at the early treatment time point and is probably due to the RSI_3C_ classifier’s quantification reflecting tumor cellularity, rather than tumor vascular perfusion as in DCE. The RSI_3C_ classifier is based on the first two components of the RSI_3C_ model (C_1_ and C_2_), which have previously demonstrated discrimination of cancer from healthy breast tissue in the pre-treatment setting (16). This is likely due to these two components corresponding to cancer while simultaneously accounting for varying degrees of fatty tissue and fibroglandular tissue. Compared to ADC, the predictive performance of the automatic RSI_3C_ classifier was of the range of DCE at all time points. Our DCE results are consistent with another directly comparable study (11), where the longest diameter of manual DCE had an AUC of predicting pCR at the early time point of 0.64; AUC increased to 0.70 using a threshold-based DCE model (functional tumor volume, FTV) (11). RSI_3C_ in the breast is sensitive to slow diffusion within hypercellular tumors while simultaneously suppressing signal from healthy fatty and fibroglandular breast tissue (14). It is expected that the RSI_3C_ classifier reflects the decrease in cellularity through the course of neoadjuvant therapy, consistent with RSI’s known estimation of tumor cellularity (20) and Gleason grade in the prostate (21, 22), though there was only moderate correlation to cellularity in the current study. This might reflect the mechanism of action of the primary chemotherapies used in this study, taxane and anthracycline, which arrests cells in mitosis (4, 25) and thus leads to cell death. This may help resolve early-phase diagnostic challenges of tumors that regress with diffuse cell loss, observable in functional measurements such as DWI, rather than with direct tumor shrinkage, meaning little or no change in overall tumor size on DCE.

Assessment of neoadjuvant therapy response at an early time point is important for clinical decision-making, enables tailored treatment regimens, and yields valuable information on *in vivo* treatment efficacy. Thus, the current findings response assessment after only three weeks by the RSI_3C_ classifier with an accuracy of 0.76 may be of particular clinical interest. Establishing early response status may allow for non-responding patients in adaptive treatment regimens to switch to alternative treatment regimens pre-surgery. This allows for the planning of additional systemic therapy for non-responders, which is known to improve survival (29, 30). On the other hand, establishing early complete responders may facilitate de-escalated treatment strategies such as shortened treatment regimens (31), thus avoiding unnecessary chemotherapy with toxic side effects.

The RSI_3C_ classifier is also informative of tumor cellularity in later phases of therapy, which is important as post-therapy cellularity is associated with overall patient survival (29). Furthermore, the RSI_3C_ classifier identified 71% of cases demonstrating residual tumor at surgery with 90% specificity, in line with conventional manual DCE measures (65% sensitivity with 90% specificity). DCE-based methods conventionally have a prominent role in the context of surgical planning after neoadjuvant therapy. However, our results suggest that the RSI_3C_ classifier may have a role in complementing DCE in this setting.

For precise longitudinal assessment of breast tumors during neoadjuvant therapy, it is crucial for imaging methods to detect treatment-related changes in vital tumor tissue as opposed to tumor-related changes such as edema and necrosis. Necrosis was purposely left out of the cancer ROI at the pre-treatment time point but may have been included in the ROI if it developed through the subsequent time points, while edema may have been included in the ROI also at the pre-treatment time point (**Supplemental Figures 5B and 6**). As such, our study did not systematically investigate the direct influence of these tumor-related changes. Nevertheless, the limited efficacy of ADC (AUC< 0.5 for ΔADC early-, mid-, and post-therapy and AUC = 0.52 for absolute ADC post-therapy) compared to the RSI_3c_ classifier in response assessment in our study may indicate a decreased ability to evaluate treatment-related changes in tumor cellularity in the presence of concomitant changes in edema and necrosis. These effects on ADC are known to become more pronounced as the *b*-value is reduced because of greater sensitivity to the fast hindered diffusion, thereby raising the ADC (15–17). Since our dataset focused on high *b*-values acquired for RSI, only 0 and 500 s/mm^2^ were available for ADC calculation, and not a range of several low *b*-values as in comparable studies (8). This means that the possible effects of tumor-related edema and necrosis may have been more pronounced in our study.

Compared to RSI_3c_, previous studies have shown that ADC seems more sensitive to the heterogeneous breast tissue environment (14, 24), consisting of varying degrees of fatty tissue (hyper-restricted, low ADC) and fibroglandular tissue (hindered diffusion, higher ADC). This is consistent with results demonstrated in **Supplemental Figure 5A** where cancer ADC values scarcely fall below that of the contralateral healthy breast control region at the pre-treatment time point, likely due to the presence of hyper-restricted fatty tissue. As the amount of viable tumor tissue decreases as response to treatment and returns to healthy breast tissue, the ADC response seems thus partly driven by the characteristics of the background tissue rather than by the treatment-related changes in tumor cellularity. This means that ADC may have a “paradoxical” response pattern where ADC decreases in a responding case as it returns to background fatty tissue with lower ADC than the original tumor ADC (**Supplemental Figure 6**). This is another factor that may explain why ADC decreased with response, resulting in the unanticipated AUC values below 0.5, contrary to our *a priori* assumption based on previous studies that an increase in mean ADC represents response to treatment (8–13). RSI_3c_ is on the other hand less influenced by normal background tissue (14, 24). These effects on ADC are likely to have been enhanced by the ROI delineation method in our study, where the pre-treatment cancer ROI was used for all subsequent longitudinally registered time points. This is different from conventional ADC, where cancer ROIs typically are manually defined for each time point (6), avoiding the inclusion of any healthy tissue.

Despite many studies examining the role of ADC in neoadjuvant therapy response assessment, there are conflicting results in the literature. Although several single-center studies have found ADC to predict response also in the early phase (10, 12) (13), the multi-center ACRIN 6698 trial (8) and a recent study by Almutlaq et al. (9) show a low predictive value of ADC at this time point, although predictive at the mid- and post-treatment time point in the ACRIN 6698 trial (8). These inconsistent findings may reflect the high sensitivity of ADC to healthy background tissue, tumor-related edema, and necrosis, which again are influenced by ROI methodology and *b*-value selection. As such, the methodological factors in our ADC analysis, differing from more conventional ADC analysis, indicate that it is difficult to determine if the RSI_3c_ classifier performs better than conventional ADC based on our findings.

There were some limitations to our study. Most notably, the sample size of this longitudinal study was small. A total of 27 patients were included, where 17 had all four MRI scans in the study protocol, which limits the reliability and generalisability of the conclusions. One limitation of the RSI_3C_ classifier method is the remaining requirement for user input for generating the pre-treatment ROI, although tumors that receive neoadjuvant chemotherapy are generally large (> 4 cm) and relatively easy to detect on pre-treatment MRI scans, whereas the more challenging task of delineating tumors that are affected by treatment is avoided. We also acknowledge that the current study is an initial application of the RSI_3C_ classifier in a neoadjuvant breast cancer setting; further optimization of the methodology (i.e. threshold value of RSI_3C_ map, cancer ROI expansion) is an area of interest for future research. Additionally, the current registration method used in the study (FLIRE) may introduce artificial changes in tumor size across time (**Supplemental Figures 1–4**) as this approach is applied to the whole breast and not specifically to the tumor. However, the focus in the current paper was on characterizing changes in tissue properties, using RSI_3C_-based measures, within the tumor across time. Lastly, as discussed above, the ADC value appeared to associate with a decrease in ADC with response; this finding was unexpected, and possibly methodological rather than physiological in origin, and should be investigated further in a larger cohort with greater statistical power.

It is well-known that ADC has a comparable ability to DCE in discriminating between benign and malignant lesions in pre-defined small regions of the breast (32, 33). Nonetheless, images that exhibit strong contrast between the tumor and surrounding healthy tissue are imperative to enhance the clinical utility of DWI. This is relevant in a range of settings including early detection, treatment evaluation, surgical planning, and surveillance. Previous studies have demonstrated that RSI_3C_ improves tumor conspicuity and tumor discrimination from healthy breast tissue compared to ADC (14, 24), which suggests that RSI_3C_ can increase the current role of DWI in clinical settings. In the current study, the RSI_3C_ classifier can automatically estimate tumor volume following a single cancer ROI definition, which is an advantage compared to conventional DWI which requires manually defined regions for every time point during neoadjuvant therapy. This may improve clinical decision-making to enable tailored treatment regimens. Automatic assessment is particularly useful in the treatment setting, as defining tumor volume is especially difficult when the tumor shrinks in size and may be affected by treatment- and procedure-related changes. RSI_3C_ may also enable non-contrast MRI, which could allow for increased monitoring of response by avoiding time and costs related to administering Gadolinium-based exogenous contrast agents. RSI_3C_ may also aid in other areas of patient care such as guiding biopsies to the most cellular aspects of a patient’s tumor for improved diagnosis and treatment planning. However, further research is necessary to assess the advantages of RSI_3C_.

The development of advanced DWI methods such as RSI_3c_ lays the foundation for a quantitative, easily implemented, and cost-efficient framework for clinical use. The multi-component RSI_3c_ model uses globally determined, fixed component ADCs, thereby enabling rapid fitting (34) of the diffusion signal making it suitable for application as a turn-key processing stream on multiple MRI platforms (14). Acquisition-wise RSI_3c_ uses high *b*-value ranges (up to 4000 s/mm^2^ in this study with a scan time of 4 minutes and 25 seconds) which only requires simple modifications to clinical breast MRI protocols which typically include low- and mid *b*-values (500-1000 s/mm^2^). Furthermore, in the neoadjuvant therapy setting, RSI_3C_ uses an effective rapid longitudinal registration (26) incorporating the pre-treatment MRI scan which automates the response evaluation and requires minimal user input. These factors are important for implementing RSI_3C_ in standard-of-care breast MRI.

Compared to DCE, DWI is prone to several image quality issues such as low signal-to-noise ratio, spatial resolution, and B_0_ inhomogeneities in EPI acquisitions which can cause warping. Specifically in the screening setting, spatial resolution is important; while DCE has excellent spatial resolution, it is limited in standard DWI. It is therefore likely that RSI_3C_, as well as conventional ADC, will miss small malignant lesions (≤ 12 mm in size), which is a well-known limitation for breast DWI (35–37). Improvement of these limitations is important for DWI to act as a reliable diagnostic tool.

In conclusion, our study demonstrates that the RSI_3C_ classifier, an automatic quantification procedure based on the three-component RSI DWI model using elastically-registered images, showed promising ability to assess response to treatment after only three weeks of neoadjuvant breast cancer therapy. The classifier eliminates the need for pre-defined lesions for each imaging time point that is required for conventional DWI and DCE analysis. We propose the RSI_3C_ classifier as a novel response biomarker that can work as a diagnostic tool in both early and late-phase of treatment. The RSI_3C_ classifier shows highly promising diagnostic properties which warrant large-scale validation studies in routine breast cancer detection and follow-up in comparison to DCE and ADC metrics.

## Data availability statement

The data analyzed in this study is subject to the following licenses/restrictions: Not approved for release beyond university hospital. Requests to access these datasets should be directed to rrakowpenner@health.ucsd.edu.

## Ethics statement

The studies involving humans were approved by University of California San Diego IRB. The studies were conducted in accordance with the local legislation and institutional requirements. The participants provided their written informed consent to participate in this study.

## Author contributions

RRP contributed to the conceptualization of the study, and together with TFB, AMD and NPJ contributed with supervision. AMD, RRP, MWT, LF, SL and AERS contributed with data curation, software development and methodology. MMSA performed formal analysis, data curation, wrote the first draft of the manuscript and contributed with methodology. AW, SZ, HO-F, JK and MH contributed with resources. TMS helped with methodology. RRP also contributed with funding resources and project management, and expert radiology analysis. All authors read, edited and approved the submitted version of the manuscript.
