# Supplementary material for: Restriction spectrum imaging with elastic image registration for automated evaluation of response to neoadjuvant therapy in breast cancer
Source: Front Oncol. 2023 Sep 15;13:1237720. doi: 10.3389/fonc.2023.1237720 (PMC10541212; doi:10.3389/fonc.2023.1237720)
Supplement: Supplementary file 1 [file DataSheet_1.zip › Image 5.PDF]

Supplemental Figure 5:

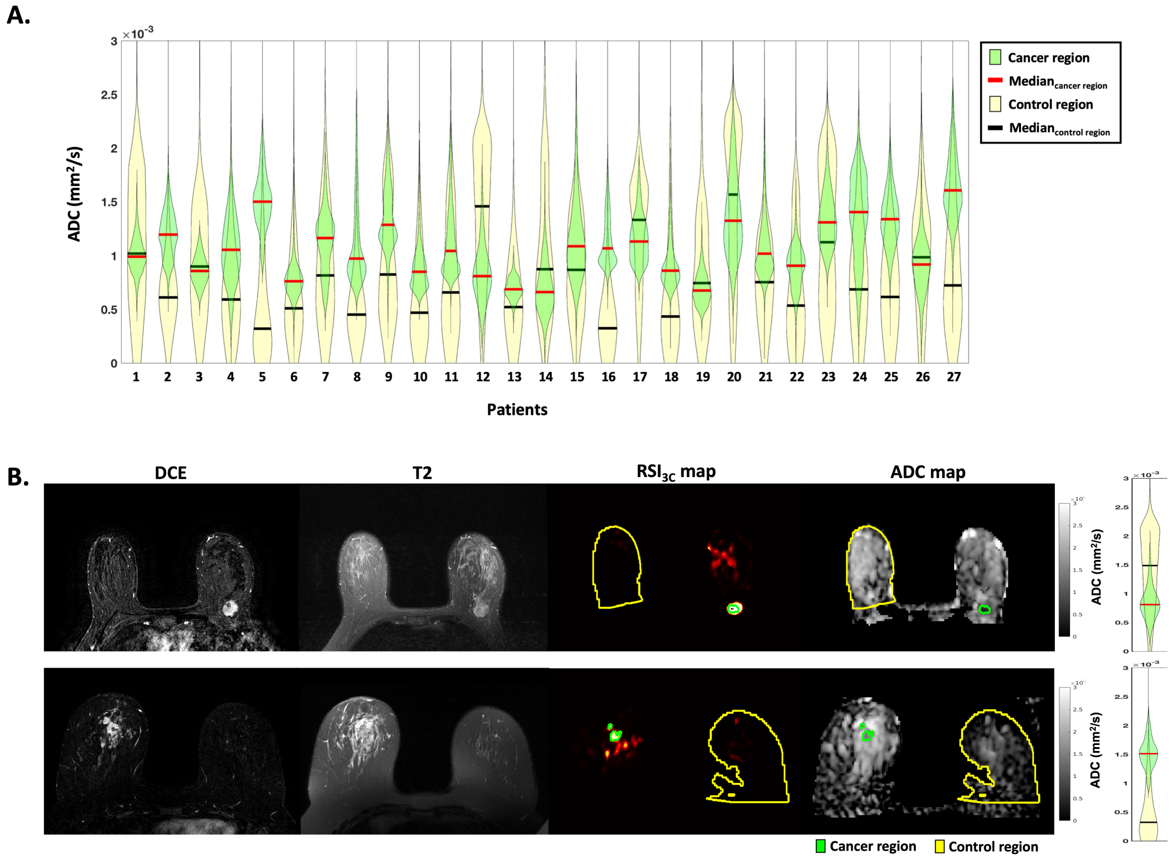

Supplemental Figure 5: A. Violin-plot showing the distribution of ADC values from cancer (green) and control (yellow, contralateral healthy breast tissue) for all patients at the pre-treatment time point (n=27). On the violin-plot, the central mark is the median for the cancer region (red) and control region (control). Note that cancer ADC values are in general higher than the control region. **B.** DCE, T2, RSI_{3C}, and ADC map with cancer (green) and control (yellow, contralateral healthy breast tissue) region with corresponding violin-plot showing the distribution of ADC values from cancer and control region. First row shows a case (patient 12 from plot shown in A) where the cancer region is lower than that of the control region, likely due to the presence of abundant fibrogladular healthy tissue in the control region. Second row shows a case (patient 5 from plot shown in A) where ADC of cancer is distinctly higher than that of the control region, which may represent inclusion of edema in the cancer region and presence of abundant fatty tissue in the control region.

DCE = dynamic contrast-enhanced MRI, RSI_{3C} = three-component Restriction Spectrum Imaging model, ADC = apparent diffusion coefficient.
