# Supplementary material for: Restriction spectrum imaging with elastic image registration for automated evaluation of response to neoadjuvant therapy in breast cancer
Source: Front Oncol. 2023 Sep 15;13:1237720. doi: 10.3389/fonc.2023.1237720 (PMC10541212; doi:10.3389/fonc.2023.1237720)
Supplement: Supplementary file 1 [file DataSheet_1.zip › Image 6.PDF]

Supplemental Figure 6

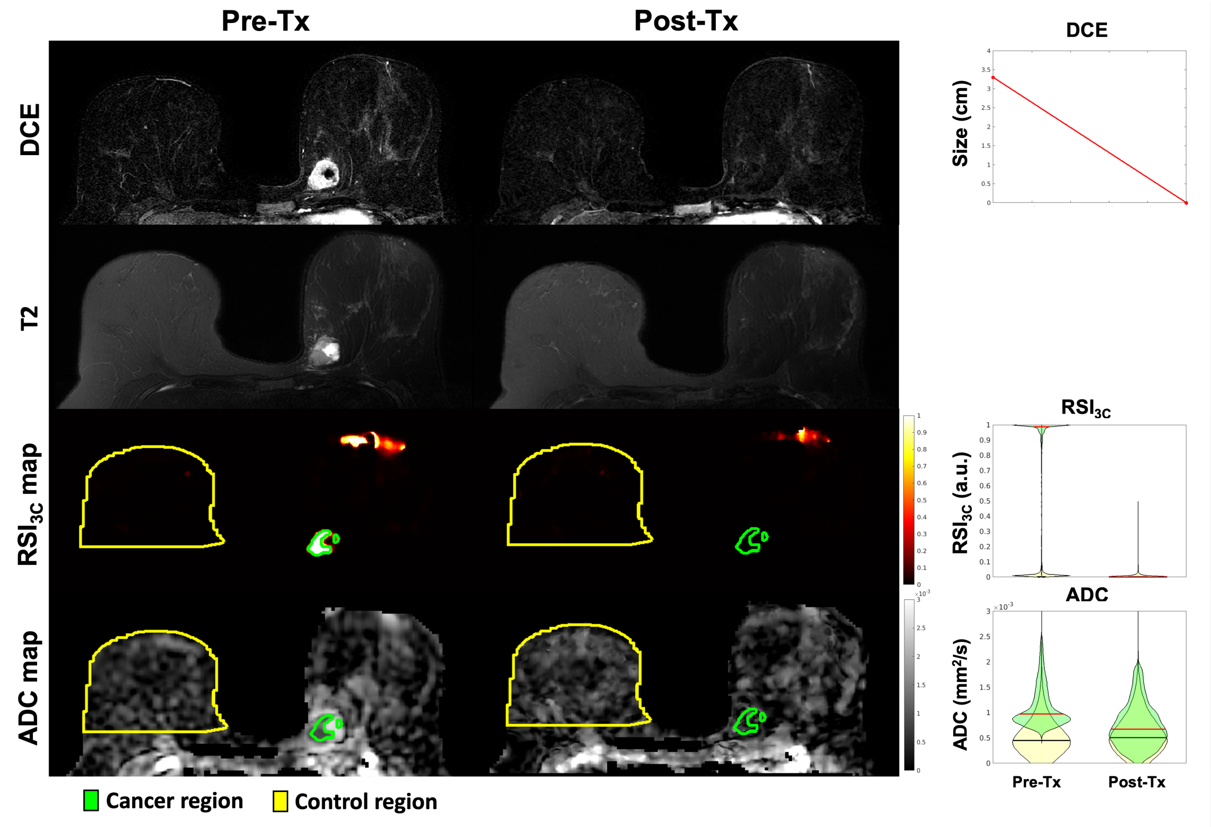

Supplemental Figure 6: DCE images with corresponding manual size measurements, T2, RSI_{3C} and ADC maps with cancer (green) and control (yellow, contralateral healthy breast tissue) region with corresponding violin-plot showing the distribution of ADC and RSI_{3C} values from cancer and control region for pre- and post-Tx time point for a subject with no remaining tumor tissue on final post-surgical pathology. Notice that for ADC at the pre-Tx time point, ADC of cancer is higher than that of the contralateral healthy breast tissue control, likely due to the presence of edema in the cancer region and abundant fatty tissue in the control region. As the amount of viable tumor tissue decreases as response to treatment and returns to healthy breast tissue, as well as decrease of edema, ADC thus exhibits a "paradoxical" response pattern where ADC decreases in a responding case as it returns to background tissue with lower ADC than the pre-Tx tumor ADC. This is different from RSI_{3C} which is less sensitive to cancer-related edema and normal background tissue (control distribution is close to zero and cancer close to one) and displays an expected response pattern where RSI_{3C} collapses to all zero.

Tx = treatment, DCE = dynamic contrast-enhanced MRI, RSI_{3C} = three-component Restriction Spectrum Imaging model, ADC = apparent diffusion coefficient.
