# Supplementary material for: Restriction spectrum imaging with elastic image registration for automated evaluation of response to neoadjuvant therapy in breast cancer
Source: Front Oncol. 2023 Sep 15;13:1237720. doi: 10.3389/fonc.2023.1237720 (PMC10541212; doi:10.3389/fonc.2023.1237720)
Supplement: Supplementary file 1 [file DataSheet_1.zip › Image 7.pdf]

Supplemental Figure 7

Receiver operating characteristics (ROC) area under the curve (AUC) for prediction of non-pCR for manual dynamic contrast-enhanced MRI (DCE), three-component Restriction Spectrum Imaging model (RSI_{3C}) classifier and the mean apparent diffusion coefficient (ADC) after all neoadjuvant therapy prior to surgical intervention (post-Tx time point).

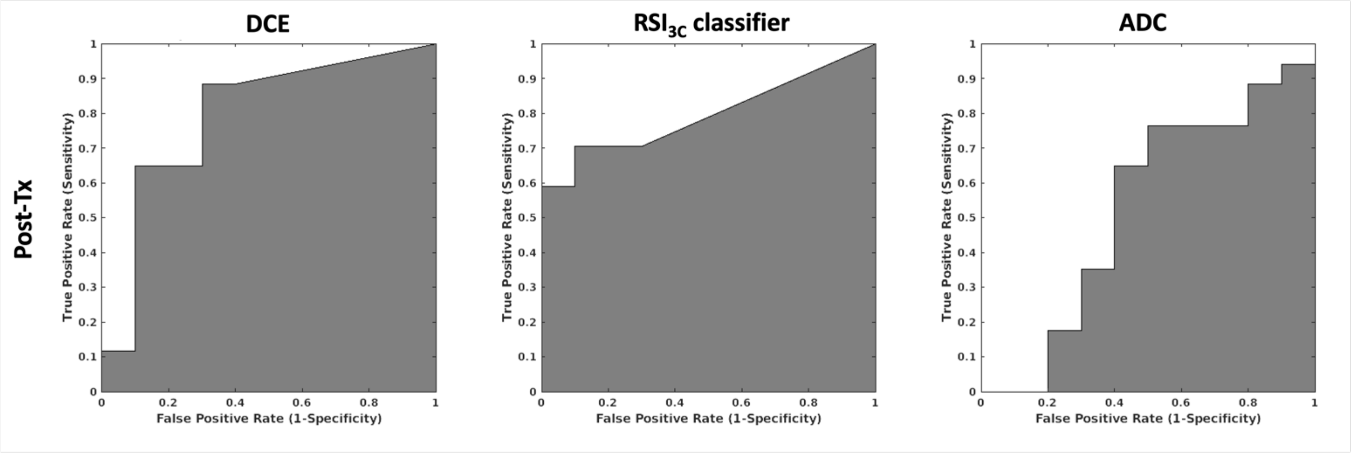

pCR = pathological complete response, Tx = treatment.
