# Supplementary material for: Restriction spectrum imaging with elastic image registration for automated evaluation of response to neoadjuvant therapy in breast cancer
Source: Front Oncol. 2023 Sep 15;13:1237720. doi: 10.3389/fonc.2023.1237720 (PMC10541212; doi:10.3389/fonc.2023.1237720)
Supplement: Supplementary file 1 [file DataSheet_1.zip › Image 8.PDF]

Supplemental Figure 8

Receiver operating characteristics (ROC) area under the curve (AUC) for the performance of ΔDCE, ΔRSI_{3C}, and ΔADC for prediction of non-pCR at each time point.

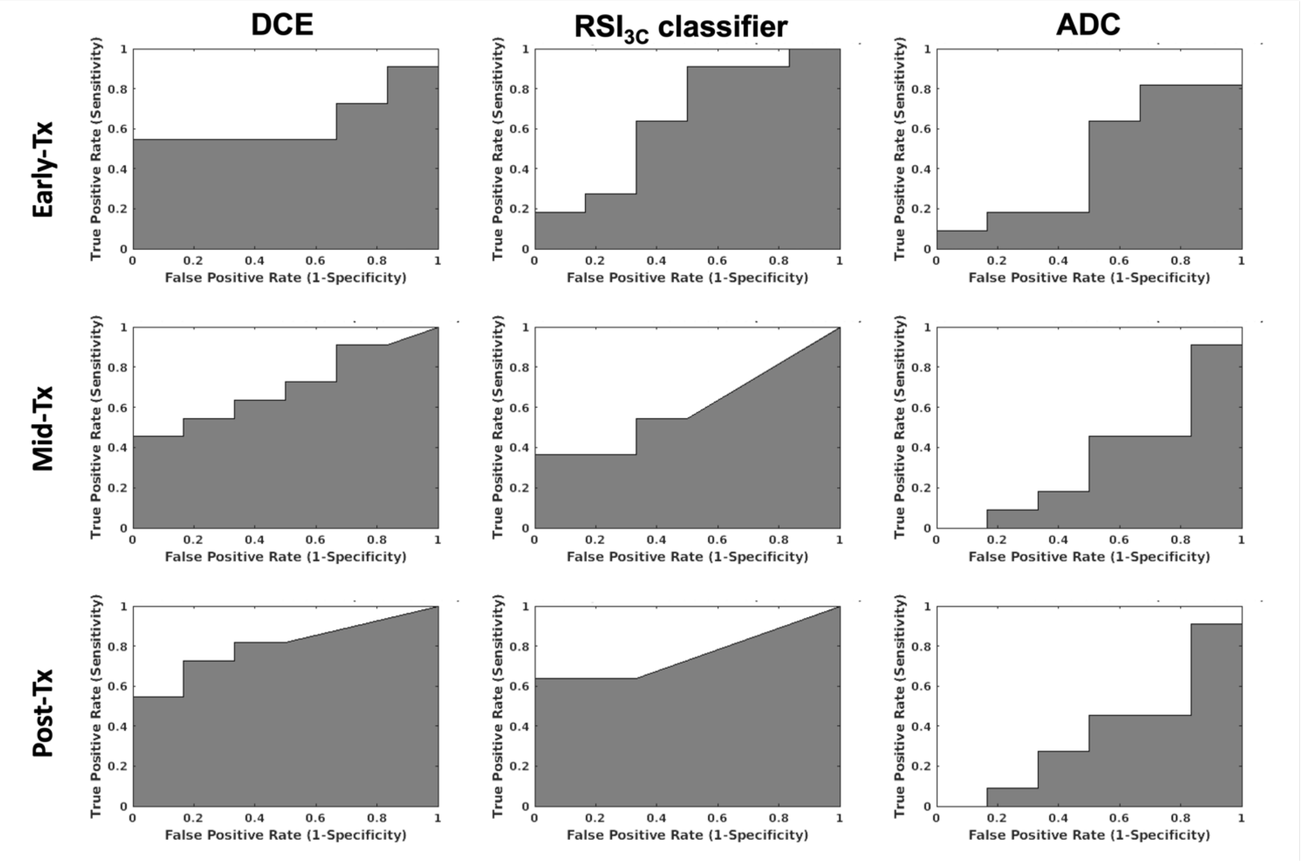

pCR = pathological complete response, Tx = treatment, ΔDCE = change in size from pre-treatment time point for manual dynamic contrast-enhanced MRI, ΔRSI_{3C} = change in size from pre-treatment time point for the three-component Restriction Spectrum Imaging model classifier, ΔADC = change in mean value from pre-treatment time point for apparent diffusion coefficient.
