# Supplementary material for: Restriction spectrum imaging with elastic image registration for automated evaluation of response to neoadjuvant therapy in breast cancer
Source: Front Oncol. 2023 Sep 15;13:1237720. doi: 10.3389/fonc.2023.1237720 (PMC10541212; doi:10.3389/fonc.2023.1237720)
Supplement: Supplementary file 1 [file DataSheet_1.zip › Image 9.PDF]

Supplementary Figure 9:

Relationship between tumor size by RSI_{3C} classifier (r = 0.6330) and DCE (r = 0.6324) and cellularity.

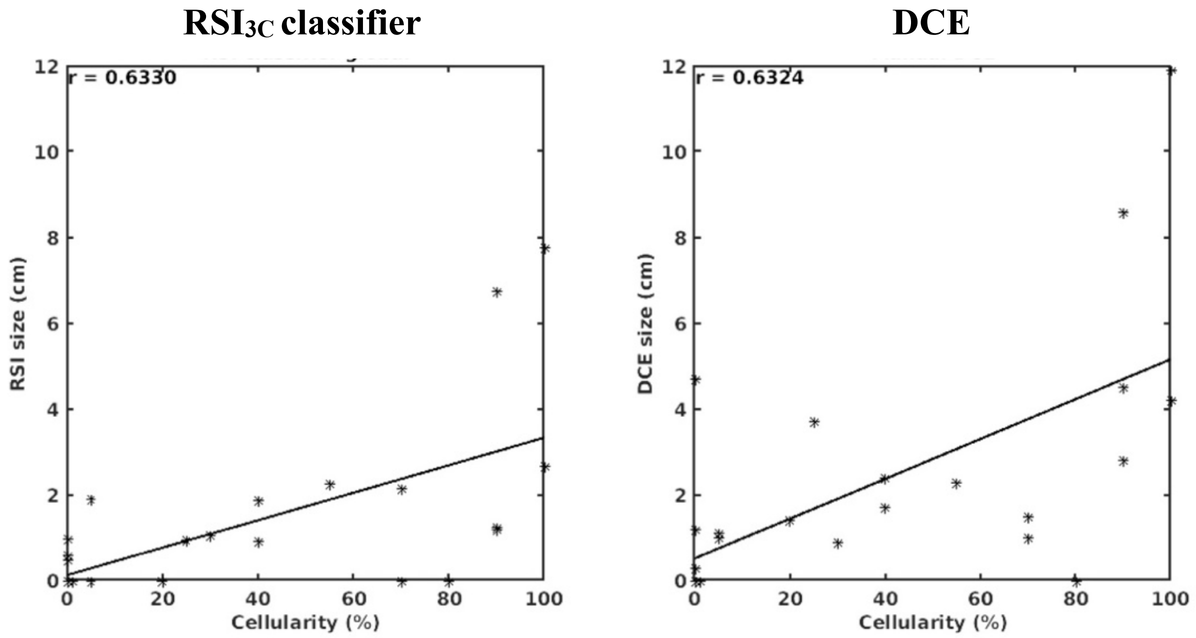

DCE = dynamic contrast-enhanced MRI, RSI_{3C} = three-component Restriction Spectrum Imaging model
