# Supplementary material for: Restriction spectrum imaging with elastic image registration for automated evaluation of response to neoadjuvant therapy in breast cancer
Source: Front Oncol. 2023 Sep 15;13:1237720. doi: 10.3389/fonc.2023.1237720 (PMC10541212; doi:10.3389/fonc.2023.1237720)

Supplemental Figure 1: Case showing a large tumor at baseline (>7cm) with response to treatment with some remaining tumor left on mid- and post-treatment scan (red arrow, 1.2 cm post-treatment). On unregistered images (top row) the breast shrinks because of massive tumor response. This introduces some geometric changes in the breast on registered (bottom row) images but has little effect on tumor size.

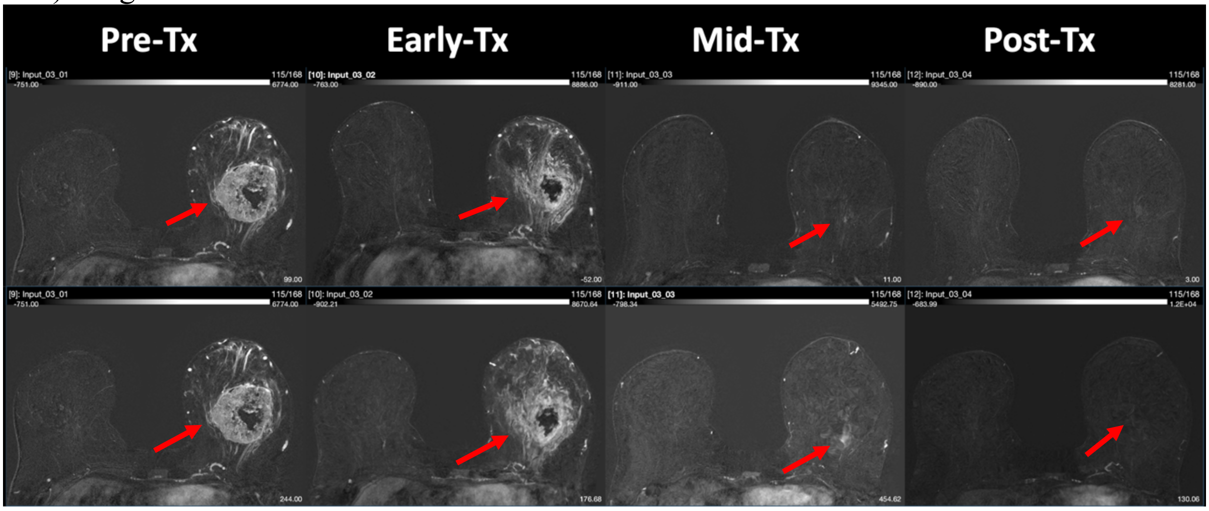

Supplemental Figure 2: Case displaying complete tumor response and no visible tumor tissue on mid- and post-treatment timepoint. Visually there is little difference in breast and tumor geometry between unregistered (top row) and registered images (bottom row).

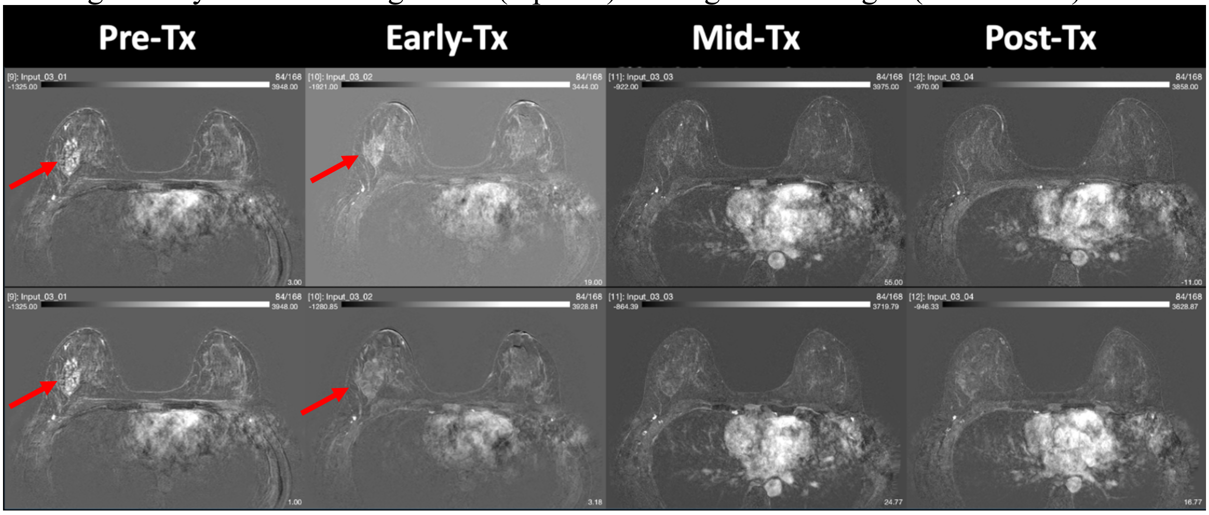

Supplemental Figure 3: Case displaying tumor with partial response to therapy with remaining 1 cm on mid- and post-treatment time points. Notice that for the mid-treatment time point, the tumor is widened in the transverse axis in the registered image (bottom row) compared to unregistered images (top row).

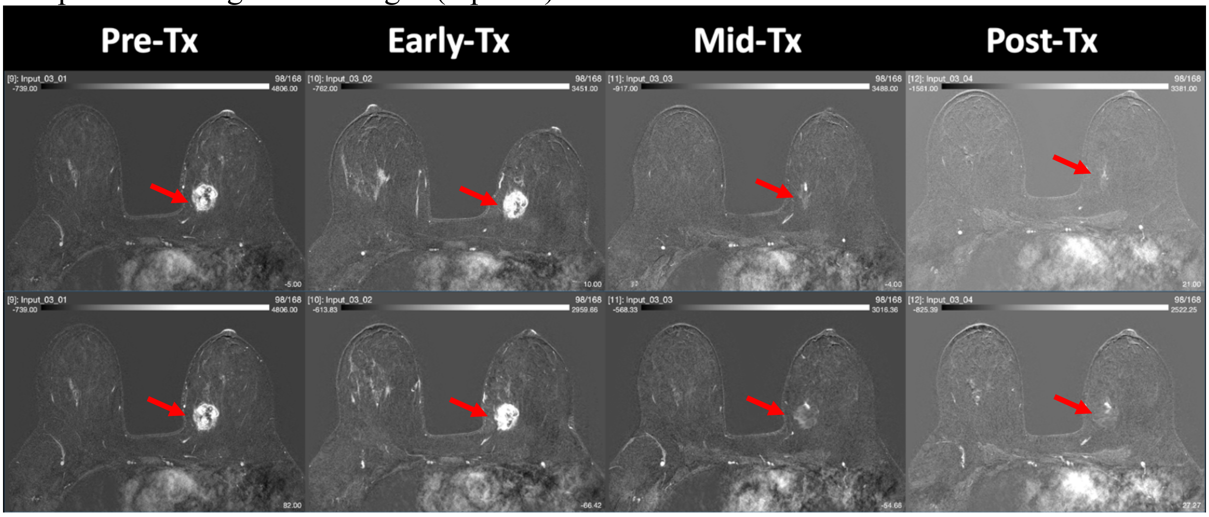

Supplemental Figure 4: Case displaying progression of disease. Notice that for the post-treatment time point, the breast increases in size along with the progression of disease in the unregistered images (top row), while the size of the breast in the registered images (bottom row) remains similar to the pre-treatment timepoint.

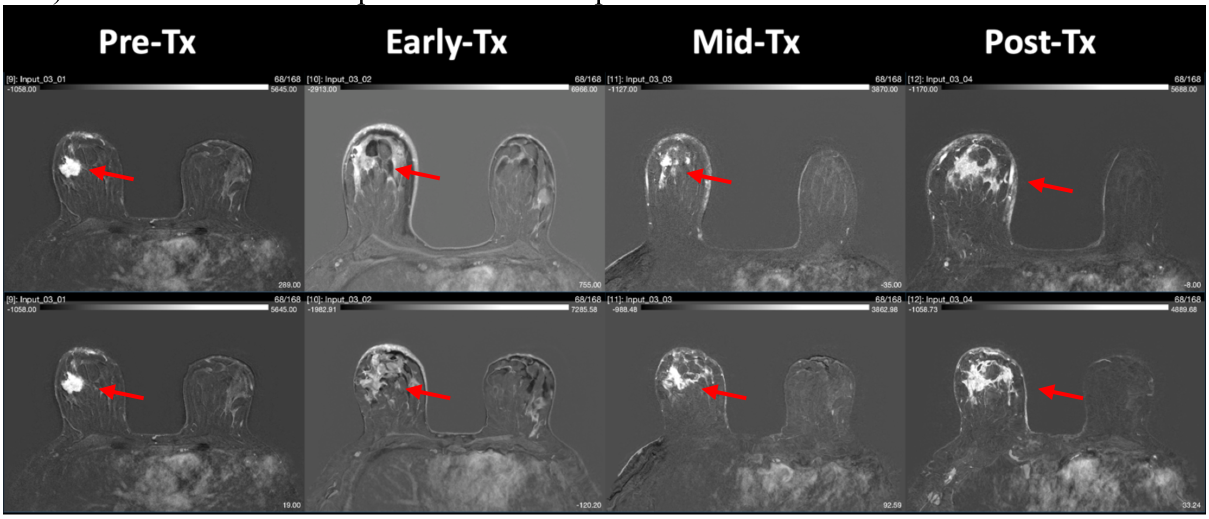

Supplement: Supplementary file 1 [file DataSheet_1.zip › Images 1-4.PDF]
