# Supplementary material for: Restriction spectrum imaging with elastic image registration for automated evaluation of response to neoadjuvant therapy in breast cancer
Source: Front Oncol. 2023 Sep 15;13:1237720. doi: 10.3389/fonc.2023.1237720 (PMC10541212; doi:10.3389/fonc.2023.1237720)
Supplement: Supplementary file 2 [file Presentation_1.pdf]

Supplementary Methods

We here give a detailed description of the methodology to calculate the longest tumor diameter by RSI_{3C} in MATLAB 2020b. The longest tumor dimension was calculated within the tumor-containing region, which was determined as described in methods section in the main portion of the manuscript. After the tumor-containing region was determined, the voxels were resampled to an isotropic resolution to facilitate dimension measurements. Then, a 0.5 threshold was applied. After this, the 'regionprops3' function was applied to derive the length (in voxels) of the major axis of the largest connected component by using the field 'PrincipalAxisLength'. The length was then converted to cm by multiplying by in-plane voxel size (2.5 mm).
