# Supplementary material for: Restriction spectrum imaging with elastic image registration for automated evaluation of response to neoadjuvant therapy in breast cancer
Source: Front Oncol. 2023 Sep 15;13:1237720. doi: 10.3389/fonc.2023.1237720 (PMC10541212; doi:10.3389/fonc.2023.1237720)
Supplement: Supplementary file 3 [file Table_1.pdf]

Supplemental Table 1

Sensitivity and accuracy given specificity ≥ 90% and receiver operating characteristics (ROC) area under the curve (AUC) for prediction of non-pCR for manual dynamic contrast-enhanced MRI (DCE), three-component Restriction Spectrum Imaging model (RSI_{3C}) classifier and the mean apparent diffusion coefficient (ADC) after all neoadjuvant therapy prior to surgical intervention (post-Tx time point).

	**DCE**	**RSI_{3C}**	**ADC**
Threshold value	1.3 cm	0.75 cm	0.5×10^{-3} mm^2/s
Specificity threshold	0.90	0.90	1.00
$Sens_{90}$ (95% CI) Post-Tx	0.65 (0.38-0.86)	0.71 (0.44-0.90)	0.00 (0.00-0.20)*
Acc_{90} (95% CI) Post-Tx	0.74 (0.54-0.89)	0.78 (0.58-0.91)	0.37 (0.19-0.58)*
ROC AUC	0.79	0.80	0.52

**Specificity ≥ 90% is achieved by a threshold where all cases are classified as pCR (specificity = 100%). For reference, sensitivity was 0.18 and accuracy 0.41 when using a specificity ≥ 80%.*

pCR = pathological complete response, $Sens_{90}$ = sensitivity given specificity ≥ 90%, Acc_{90} = accuracy given specificity ≥ 90%, Tx = treatment.
