# Supplementary material for: Restriction spectrum imaging with elastic image registration for automated evaluation of response to neoadjuvant therapy in breast cancer
Source: Front Oncol. 2023 Sep 15;13:1237720. doi: 10.3389/fonc.2023.1237720 (PMC10541212; doi:10.3389/fonc.2023.1237720)
Supplement: Supplementary file 4 [file Table_2.pdf]

Supplemental Table 2

Sensitivity and accuracy given specificity ≥ 90% and receiver operating characteristics (ROC) area under the curve (AUC) for performance of ΔDCE, ΔRSI_{3C} and ΔADC for prediction of non-pCR at each time point. Since there was no threshold value yielding specificity = 90% for any modality at any time point, the closest threshold of specificity = 100% was used. Note that post-Tx time point is after all neoadjuvant therapy prior to surgical intervention. Threshold values are unitless multiplication factors.

		**ΔDCE**	**ΔRSI_{3C}**	**ΔADC (↑ = response)**
Early-Tx (3 weeks)	AUC (95%CI)	0.64 (0.36-0.91)	0.65 (0.38-0.92)	0.45 (0.16-0.75)
	$Sens_{90}$	0.55	0.18	0.09
	Acc_{90}	0.71	0.47	0.41
	Thresh.	-0.07	0.12	-0.17
Mid-Tx (12 weeks)	AUC (95%CI)	0.71 (0.45-0.96)	0.60 (0.32-0.88)	0.35 (0.06-0.64)
	$Sens_{90}$	0.46	0.36	0.00*
	Acc_{90}	0.65	0.59	0.35*
	Thresh.	-0.23	-0.15	-0.77
Post-Tx	AUC (95%CI)	0.80 (0.59-1.00)	0.76 (0.52-0.99)	0.36 (0.07-0.65)
	$Sens_{90}$	0.55	0.64	0.00*
	Acc_{90}	0.71	0.76	0.35*
	Thresh.	-0.48	-0.81	-0.73

**Specificity ≥ 90% is achieved by a threshold where all cases are classified as pCR (specificity = 100%). For reference, sensitivity was 0.09 and accuracy 0.35 when using a specificity ≥ 80%.*

pCR = pathological complete response, $Sens_{90}$ = sensitivity given specificity ≥ 90%, Acc_{90} = accuracy given specificity ≥ 90%, Tx = treatment, ΔDCE = change in size from pre-treatment time point for manual dynamic contrast-enhanced MRI, ΔRSI_{3C} = change in size from pre-treatment time point for the three-component Restriction Spectrum Imaging model classifier, ΔADC = change in mean value from pre-treatment time point for apparent diffusion coefficient.
